# Accuracy of digital surgical guides for dental implants

**DOI:** 10.1186/s40902-022-00364-4

**Published:** 2022-10-25

**Authors:** Myoung-Ju Kim, Jun Young Jeong, Jaeyoung Ryu, Seunggon Jung, Hong-Ju Park, Hee-Kyun Oh, Min-Suk Kook

**Affiliations:** Department of Oral and Maxillofacial Surgery, School of Dentistry, Dental Science Research Institute, Chonnam National University, 33 Yongbong-ro, Buk-Gu, Gwangju, Republic of Korea 61186

**Keywords:** Digital implant surgery, Digital surgical guide, Accuracy, Dental implants, R2GATE

## Abstract

**Background:**

Recently developed imaging techniques, such as cone beam computed tomography (CBCT) and CAD/CAM technology, have facilitated reliable implant planning and implant surgical guide production by 3D printing. This study compared the accuracy of implant-guided surgery using the R2GATE^®^ program with CBCT before and after surgery.

**Patients and methods:**

The study included patients who visited the Department of Oral and Maxillofacial Surgery at Chonnam National University Hospital from September 2021 to March 2022. Twenty-four implants were placed in eleven patients. Using R2GATE^®^ Windows (Megagen implant, Daegu, Korea) software, implant placement was planned. The difference was measured by the CBCT before and after surgery. The cervical and apical distance and angular deviation of the implants were measured. Statistical analysis was performed using an independent t-test, Pearson correlation, and multiple regression analyses.

**Results:**

The three-dimensional linear distance difference between the planned implant and the placed implant was 0.97 ± 0.37 mm at the cervical and 1.13 ± 0.36 mm at the apical. The difference in angle deviation between the planned implant and the placed implant was 3.42 ± 2.12°. Among the variables affecting the accuracy of implant placement, a statistically significant difference was found when using a tissue-supported implant guide, implant diameter and implant length.

**Conclusion:**

Based on these results, using the R2GATE^®^ program is useful to use an implant digital surgical guide, and it will be used in various clinic.

## Background

Implant surgical guide guides facilitate proper positioning and angulation of implants using cone beam computed tomography (CBCT) and assist in treatment planning [1].

Although traditional plaster model-based implant surgical guides have been produced for a long time, recent imaging techniques, such as CBCT, provide a three-dimensional evaluation of various anatomical structures, such as residual bone and nerves, before implant surgery. Moreover, with the recent introduction of CBCT, it is possible to obtain high-quality images with reduced radiation exposure to patients [2]. CBCT enables a three-dimensional reconstruction of the patient’s oral cavity. With the development of CAD/CAM (Computer-Aided Design/Computer-Assisted Manufacturing) technology, 3D printing digital data allows clinicians to perform 3D implant simulations and fabricate digital surgical guides for clinical practice [3].

Clinicians can integrate imaging data with implant placement planning and 3D-printed surgical guides to manage implant challenges. Several studies have reported high accuracy for implant surgery performed with surgical guides [4].

Implant surgical guides can assist in accurate implant placement according to the treatment plan and simplify surgical procedures [5]. Moreover, minimally invasive surgery is achieved by 3D implant software that provides a better understanding of patient anatomy before surgery [6]. CBCT-driven implant planning uses a top-down approach to determine the final shape of the prosthesis and ensure predictability in abutment angulation or positioning holes in screw-type prostheses. A treatment plan established with these considerations can be helpful to older adults or patients with systemic diseases who face the challenges of long-term surgery or are sensitive to invasive procedures. Furthermore, the possibility of immediate provisional restoration fabrication enables esthetic rehabilitation in critical cases. Thus, immediate restoration can facilitate satisfactory occlusion, load distribution, and optimal oral hygiene [7].

A disadvantage is that surgical guides supported by a few remaining teeth or soft tissue may exhibit poor stability during implantation. Moreover, reduced mouth opening may limit the accessibility of surgical guides during implant placement in the posterior region [8]. The operator should recognize the existence of a learning curve for familiarization with surgical guides and guide drill systems before applying the procedure [9]. Surgical guides can achieve precision in implant insertion compared to free-hand surgery, resulting in a lower potential for deviation, higher implant stability, and fewer errors in the manufacturing process.

This study used, among various implant simulation software, the R2GATE^®^ program to establish a surgical plan and evaluate the accuracy of 3D-printed implant surgical guides in patients who underwent implant surgery in the hospital.

## Patients and method

### Patients

The study included patients who visited the Department of Oral and Maxillofacial Surgery at Chonnam National University Hospital from September 2021 to March 2022 for dental implant treatment due to tooth loss. The researchers provided study-related information to the patients and obtained voluntary informed consent to participate in the study. Patients were selected through screening by evaluating for systemic diseases at the first visit. There are systemic diseases such as the cardiovascular system, digestive system, respiratory system, endocrine system, central nervous system, or mental illness that could significantly influence this clinical trial, or bone graft or implant experience in the past, history of tumor removal, presence of osteomyelitis, maxillary sinusitis or uncontrolled periodontal disease, oral radiation therapy in the past, inadequate oral hygiene management skills, and mouth opening limitation due to temporomandibular joint disease were excluded.

### Surgical guide preparation

All patients included in the study were subjected to panoramic radiographs and CBCT before implant placement. The plaster model produced by taking intraoral impressions was scanned using an intraoral dental scanner (TRIOS™, 3Shape, Inc., Copenhagen, Denmark). CBCT data was saved in DICOM format, and the scanned plaster model was saved in stereolithography (STL) format, and the two files were superimposed by importing into the implant planning software R2GATE^®^ Windows (Megagen implant, Daegu, Korea) program.

**Fig. 1:**
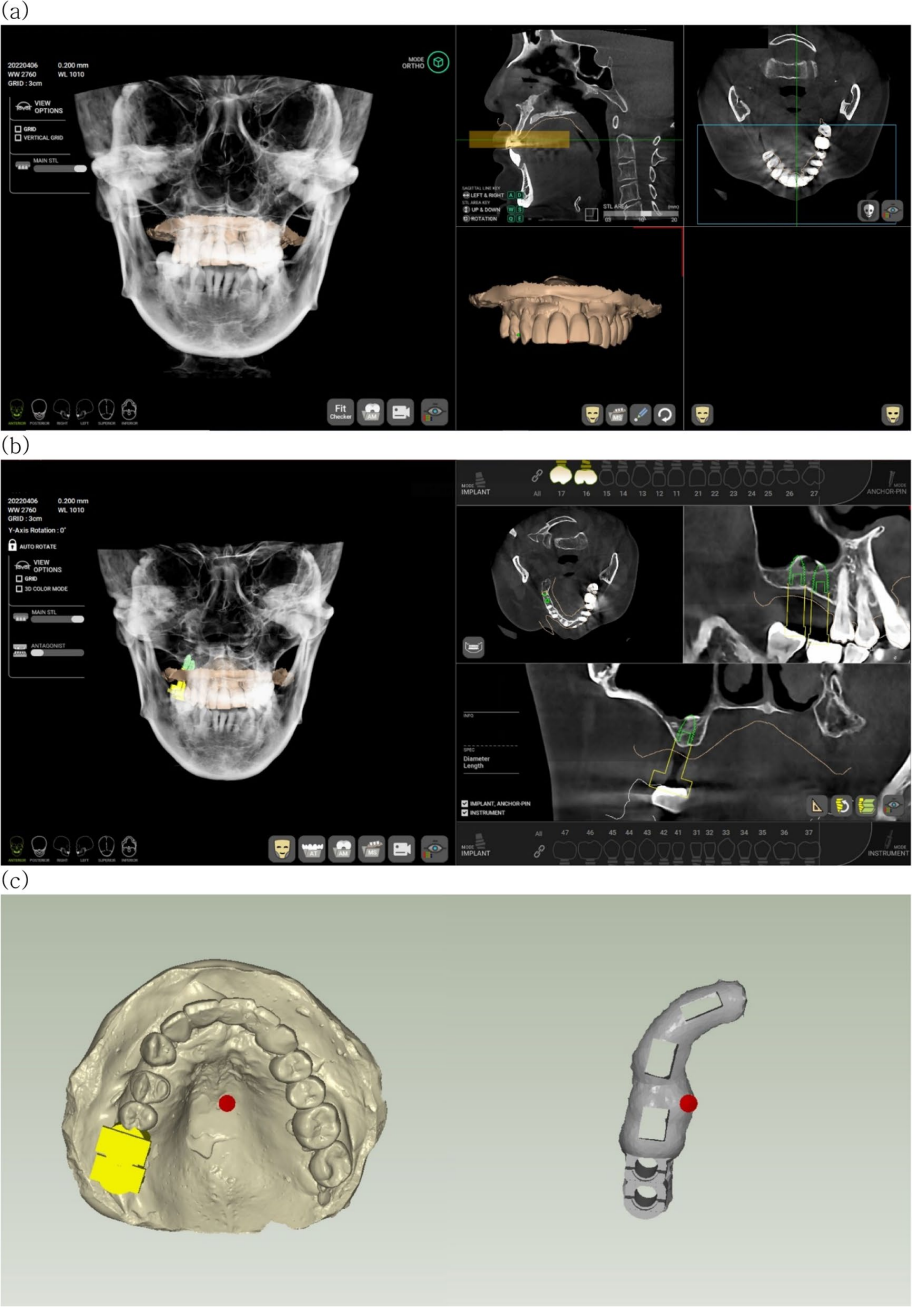
Protocol for implant surgical guide preparation using R2GATE^®^ software. **a** CBCT and STL matching. **b** Implant planning. **c** Guide design

For determining the ideal position of the prosthesis, alveolar bone density was analyzed, and an implant suitable for clinical conditions was simulated by considering adjacent anatomical structures such as the inferior alveolar nerve canal or the maxillary sinus (Fig. 1). The completed implant plan was extracted as an RWS file and imported into the R2WARE™ (Megagen implant, Daegu, Korea) program to produce an implant surgery guide. This program automatically places the guide hole at the planned implant location according to the size of the implant guide kit (provided by the manufacturer). The thickness of the guide was set to 3.0 mm, and the offset was set to 0.05 mm. The planned implant surgical guide was printed with Surgical Guide Resin using a stereolithography apparatus (SLA) type 3D printer (Form 3 + , Formlabs, Somerville, MA, USA).

### Implant surgery using surgical guide

The prepared surgical guide was adapted to the patient’s oral cavity, and the operation was performed using the R2GATE^®^ surgical kit. The drilling sequence followed the intended length and diameter of the implant. The operator confirmed initial stability in all implants immediately after placement. This study used three implant fixtures of Megagen (Megagen implant, Daegu, Korea): AnyOne^®^ internal, AnyOne^®^ external, and BLUEDIAMOND IMPLANT^®^. Among them, AnyOne^®^ internal and BLUEDIAMOND IMPLANT^®^ are internal types, and AnyOne^®^ external is external type. All surgeries were performed by experienced oral and maxillofacial surgeons.

### Analysis method

**Fig. 2:**
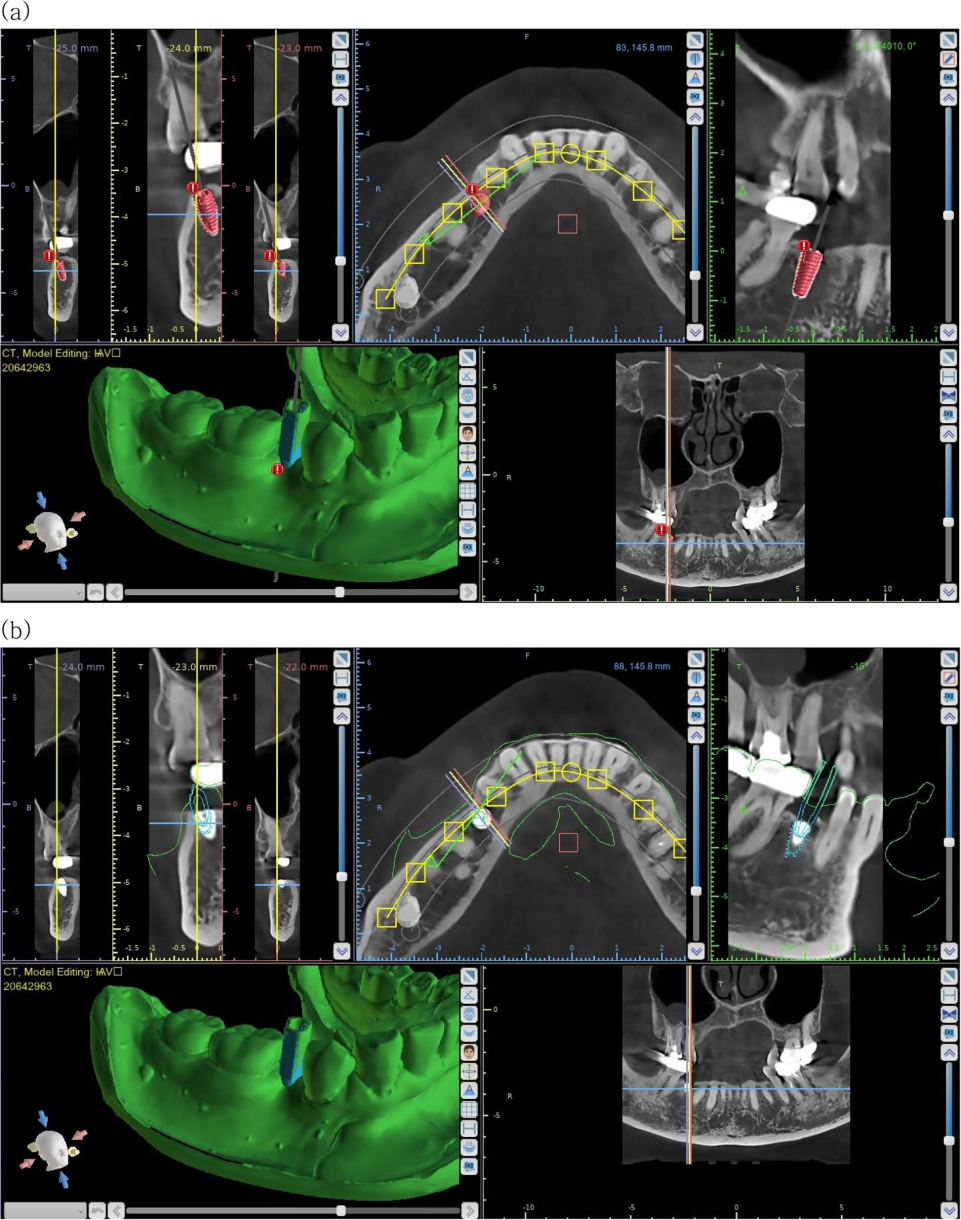
**a** Overlay fixture combined with the scan abutment on the cast with the planned implant in the Blue Sky Plan III software and extracted as an STL file. **b** The implant fixture is superimposed on the postoperative CBCT in the Blue Sky Plan III software and extracted as an STL file

With Blue Sky Plan III (Blue Sky Bio, Grayslake, IL, USA) software, the post-operative fixture was extracted as an STL file by superimposing the DICOM file of the CBCT taken after surgery, and the planned fixture was extracted as an STL file by superimposing the fixture on the CBCT on the planned implant location before surgery (Fig. 2).

**Fig. 3:**
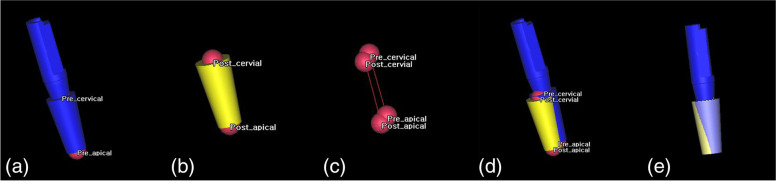
Merging implant planning STL file and postoperative placed STL file. **a** Midpoint of the planned implant. **b** Midpoint of the placed implant. **c** Each point and lines. **d** Before merging of planned and placed implant. **e** Merging state of the planned and placed implant

The extracted fixtures were superimposed on SimPlant O&O software (Materialise, Leuven, Belgium). The three-dimensional coordinates of the apical end and cervical center of the planned implant fixture and the placed implant fixture were measured (Fig. 3). The angle difference was calculated with a line connecting the center point of the apical end and the cervical region of the planned and placed implant.

### Statistical analysis

Statistical analysis was performed using the SPSS version 27 (SPSS, Chicago, IL, USA) program. Mean values were used for analysis. An independent *t*-test was used to compare the accuracy of the planned implant and placed implant positions. Pearson correlation analysis was performed to evaluate the relationship between each variable and the accuracy of implant placement. A multiple regression analysis was performed to investigate the influence of these variables on the accuracy of implant placement.

## Results

This study included 11 patients and excluded 4 patients who did not use an implant guide during fixture placement among 15 patients. The average age was 60.5 ± 12.3 years (4 males; 7 females). A total of 24 implants were placed, of which 8 used tooth-supported guides and 16 used tissue-supported guides. According to the arch, 15 implants were placed in the maxilla and 9 in the mandible. Ten implants were placed in the anterior region and 14 in the posterior region (depending on position). For implant fixtures, 14 AnyOne^®^ internal, 5 AnyOne^®^ external, and 5 BLUEDIAMOND IMPLANT^®^ were placed (Table 1).

**Table 1:** Characteristics of study

Characteristics	*n*
Total number of implants	24
Gender	
Male	4
Female	7
Average age	60.5 ± 12.3
Implant guide classification	
Tooth support	8
Tissue support	16
Jaws	
Maxilla	15
Mandible	9
Placement location	
Anterior area	10
Posterior area (premolar and molar)	14
Implant fixture type	
AnyOne^®^ internal	14
AnyOne^®^ external	5
BLUEDIAMOND IMPLANT^®^	5
Implant diameter	
Mini	5
Regular	17
Wide	2
Implant length (mm)	
7.0	1
8.5	4
10.0	12
11.5	7

The three-dimensional linear distance difference between the planned and placed implant was 0.97 ± 0.37 mm at the cervical and 1.13 ± 0.36 mm at the apical (Table 2). When the error distance is divided into *x*, *y*, and *z* axes, the error on the *z*-axis reflects the accuracy of the implantation depth. Fifteen implants were placed with a deeper than planned depth, and 9 were placed with a shallower than planned depth. The difference in angle deviation between the planned and placed implant was 3.42 ± 2.12° (Table 3).

**Table 2:** Deviation between planned and placed implant (mean ± SD)

	Coronal (mm)	Apical (mm)	95% CI	*p*
3D	0.97 ± 0.37	1.13 ± 0.36	− 0.05 ~ 0.38	0.13
*X*-axis	0.39 ± 0.34	0.60 ± 0.41	− 0.01 ~ 0.43	0.06
*Y*-axis	0.48 ± 0.33	0.54 ± 0.34	− 0.14 ~ 0.25	0.55
*Z*-axis	0.61 ± 0.33	0.61 ± 0.35	− 0.20 ~ 0.20	0.99

*SD* standard deviation, *CI* confidence interval

**Table 3:** Angular deviation between planned and placed implant (mean ± SD)

	3D
Angular deviation (°)	3.42 ± 2.12

Among the variables affecting the apical deviation, the use of tissue supported implant guides had a statistically significant effect on the difference (*p* < 0.05) (Table 4).

**Table 4:** Evaluate of the relationship between each variable and the correlation with implant placement (Pearson correlation analysis)

	Cervical	Apical
Implant guide classification (tooth support vs tissue support)	− 0.278	0.738*
Jaws (maxilla vs mandible)	0.433	− 0.202
Placement location (anterior vs posterior)	0.337	− 0.184
Implant fixture type (internal vs external)	− 0.681	− 0.450
Implant diameter	− 0.105	0.674*
Implant length	− 0.421	0.940*

^*^p < 0.05

In general, there was a significant difference in the deviation of the apical end and cervical region during implant placement according to implant diameter and length (*p* < 0.05). The statistically significant apical difference was 0.482 mm as the implant diameter increased, 0.563 mm (*p* = 0.042) in the *Y*-axis and 0.544 mm (*p* = 0.043) in the *Z*-axis (Table 5).

**Table 5:** Implant diameter and length influencing the accuracy of the implant placement in the apical region

		*B*	Standard error	β	t	*p*
3D	Constant	1.999	1.380		1.449	0.162
	Implant diameter	− 0.390	0.179	− 0.482	− 2.178	0.041^*^
	Implant length	0.076	0.077	0.218	0.985	0.336
*X*	Constant	− 0.2151	1.862		− 1.155	0.261
	Implant diameter	0.171	0.242	0.186	0.705	0.488
	Implant length	0.206	0.105	0.520	1.972	0.062
*Y*	Constant	3.072	1.516		2.026	0.056
	Implant diameter	− 0.0426	0.197	− 0.563	− 2.163	0.042^*^
	Implant length	− 0.077	0.085	− 0.234	− 0.900	0.378
*Z*	Constant	2.599	1.498		1.734	0.098
	Implant diameter	− 0.420	0.195	− 0.544	− 2.157	0.043^*^
	Implant length	− 0.025	0.084	− 0.075	− 0.297	0.769

*B* non-standardized coefficient, *β* standardized coefficient, *t* test statistic (*B*/standard error)

^*^p< 0.05

## Discussion

Recently, with the development of CAD/CAM and RP technology, the application of surgical guides in the clinic has increased. Surgical guides can enable the attending physician to transfer the virtual preoperative treatment plan to the actual surgical procedure, thereby shortening the operation time and minimizing invasiveness [8]. Nickenig et al. (2010) reported that implant placement using a template was more accurate than free placement [10]. Brief et al. (2005) stated that image-guided implant placement was more precise than free placement, but the existing free placement method provided sufficient accuracy for most clinical cases [11].

In this study, a surgical guide was produced using the scanned image of a plaster model, CBCT image, and CAD/CAM (partial digital method). The surgical guide was designed using an image superimposed on a CBCT image and a scanned image of a plaster model fabricated by taking impressions; the surgical guide was 3D printed. Most surgical guides reported in the literature have been prepared using manual methods or stereolithography. According to the data on surgical guide errors produced by these methods, an average of 1.22 mm positional displacement occurred at the top of the implant, and the resulting average angle error was 4.9° [12, 13]. In this study, a surgical guide was manufactured using the partial digital method, with an error of 0.97 mm and 1.13 mm on average at the cervical and apical end of the implant, respectively, and an average angle error of 3.42°.

Images obtained with digital intraoral scanners can be overlapped with CBCT images without using impression materials or plaster models [14], thus minimizing errors due to the deformation of impression materials and plaster during the setting and fabrication of the radiation guide [15, 16]. Accuracy can be confirmed by the fit of the surgical guide in the oral cavity; moreover, intraoral scanning reduces procedure time.

However, a plaster model allows the operator to check the fit of the surgical guide before placing it in the patient's mouth. Notably, the offset value for the surgical guide printed in this study was 0.03 mm, which was inaccurate on the plaster model. Since the offset required for each 3D printer is different, the operator should check the fit of the surgical guide at every step to avoid inaccuracies and rattling.

Although surgical guides can provide greater accuracy in implant placement, the following factors should be considered. First, surgical guides can decrease the availability of intraoral space and create challenges in placing the implant drill. Therefore, it would be desirable to use a surgical guide in a patient with sufficient mouth opening. Second, surgical guides may need modifications for posterior molars due to difficulties associated with limited accessibility for drill insertion during guided surgery. Third, surgical guide stability in the oral cavity is critical to implant success. For tissue-supporting guides in long edentulous jaws, a method for fixing the surgical guide to the edentulous jaw is necessary due to lowered surgical guide stability. In the R2GATE program, the surgical guide could be seated by applying an anchor pin and fixing it with a screw on the edentulous part for improved stability.

Previous studies using implant surgery guides reported no significant difference in accuracy between experienced and inexperienced surgeons [17]. Therefore, implant surgery using implant guides may help novice dentists with insufficient skills.

## Conclusion

Implant surgery performed with the surgical implant guide using the R2GATE^®^ program showed a linear deviation of about 1 mm and an angle deviation of about 3.4°, so it may be useful to use an implant digital surgical guide and it will be used in various clinic.

## Data Availability

Please contact the author for data requests.

## Abbreviations

CBCT: Cone beam computed tomography
CAD/CAM: Computer-Aided Design/Computer-Assisted Manufacturing
3D: 3-Dimension
STL: Stereolithography
DICOM: Digital Imaging and Communications in Medicine
SLA: Stereolithography apparatus
